# The Role of Brain Glycogen in Supporting Physiological Function

**DOI:** 10.3389/fnins.2019.01176

**Published:** 2019-11-01

**Authors:** Laura R. Rich, William Harris, Angus M. Brown

**Affiliations:** ^1^School of Life Sciences, University of Nottingham, Nottingham, United Kingdom; ^2^Department of Neurology, University of Washington, Seattle, WA, United States

**Keywords:** glucose, lactate, glycogen, optic nerve, memory

## Abstract

Glycogen is present in the mammalian brain but occurs at concentrations so low it is unlikely to act as a conventional energy reserve. Glycogen has the intriguing feature of being located exclusively in astrocytes, but its presence benefits neurones, suggesting that glycogen is metabolized to a conduit that is transported between the glia and neural elements. In the rodent optic nerve model glycogen supports axon conduction in the form of lactate to supplement axonal metabolism during aglycemia, hypoglycemia and during periods of increased energy demand under normoglycemic conditions. In the hippocampus glycogen plays a vital role in supplying the neurones with lactate during memory formation. The physiological processes that glycogen supports, such as learning and memory, imply an inclusive and vital role in supporting physiological brain functions.

## Introduction

The requirement for energy is a fundamental need that is shared by all living things (Tymoczko et al., 2015). From an evolutionary point of view the emergence of life on earth commenced with the emergence of single celled organisms in aquatic environments (Davies, 1998; Gale, 2009), although the specific location of this is contentious (Djokic et al., 2017). The predominant view is the cells evolved deep in the ocean near vents that spewed super hot liquid from ruptures in volcanic vents. The latter viewpoint is that the single celled organisms evolved in pools of mud and clay and then traveled down rivers before entering the sea (Mulkidjanian et al., 2012). This explanation is appealing as the sodium to potassium ratio is the same as occurs in the cells (Mulkidjanian et al., 2012), and complies with the Macallum theory that chemical traits of cells are more conservative than changing environments, whereas in the oceans the ratios are reversed i.e., they have high sodium and lower potassium (Gale, 2009). Whatever the origin the energy requirements of these cells was relatively simple to meet as the nutrients were available in plentiful supply thus the cells did not have to search for these nutrients. All that cells required to receive adequate nutrition was a semi permeable membrane to allow entry of the nutrients and expulsion of waste products. In single celled organism the diffusion distances were small i.e., sub-cellular. At this point in evolution glycolysis was the sole metabolic pathway that produced energy in cells (Lane, 2015). As life become more complex, and as single celled organisms evolved into multicellular organisms the utilization of energy substrate became more complex as an increasing number of cells within an organism meant that diffusion was no longer an adequate means by which to deliver nutrients, and a rudimentary system of vessels for delivery of nutrients to all cells evolved, which would later evolve into the vascular system (Boron and Boulpaep, 2009). As life forms evolved into land dwelling animals they had to evolve systems for obtaining nutrients and the gastrointestinal system evolved, where energy is consumed and chemically transformed into the constituent components of the complex foods, prior to uptake by the vascular system and delivery to the cells (Boron and Boulpaep, 2009). The increasing demand for energy resulted in the development of oxidative metabolism in which bacteria entered cells and evolved into mitochondria (Stryer, 1995), which were able to use the presence of oxygen, that had accumulated in the atmosphere as a result of the developments and multiplication of blue green algae (Gale, 2009), to increase the efficiency of the energy yield (Stryer, 1995). As mammals evolved, a class of creatures that maintains a constant body temperature usually above the ambient air temperature, the requirements of energy increased dramatically (Nagy et al., 1999). Such evolution also required the capacity for storage of energy substrate as constant food availability was unlikely and thus the feast and fast process of energy consumption evolved. As a result excess substrates in the form of fat and carbohydrates were stored as subcutaneous fat, liver and muscle glycogen respectively (Boron and Boulpaep, 2009).

## Increasing Metabolic Demand Accompanies Increased Brain Size

As mammals evolved the complexity of the brain increased in parallel, and with the evolution of humans, which commenced about 1 million years ago, the large brain, and in particular the development of a large frontal cortex, had its own unique energy requirements (Wang et al., 2008; Harris and Attwell, 2012). At the current state of human evolution the energy demands of the system may be summarized as follows. The brain has a disproportionate energy demand relative to its size. It contributes only 2% of the body weight but requires 20% of the blood flow, from which it extracts 50% of available oxygen and 20% of available glucose (Dienel, 2009). Excess glucose is stored in the liver as glycogen, which can sustain normoglycemic concentrations of glucose for up to 24 h in the absence of any food intake (Stryer, 1995). The subcutaneous fat, depending on the size of the individual, can be broken down to ketone bodies and can support brain function for weeks to months depending upon the extent of the reserves (Voet and Voet, 2011).

A consequence of the evolution of a larger brain and the associated parallel increase of information processing has meant that the energy demand has increased in a non-linear fashion, and is now described by a power function (Wang et al., 2008). The consequence of this is that modest increases in brain size throughout evolution have been accompanied by disproportionally large increases in energy demand (Wang et al., 2008). Such development could put the brain at risk from not receiving an adequate supply of nutrients. It is instructive to note that in the adult human occlusion of the carotid arteries for the briefest period of time (6 to 8 s) results in a loss of consciousness (Rossen et al., 1943). This highlights in spectacular fashion the energy substrate requirements of the brain, and how even the slightest delay in delivery of such substrates, where the energy supply can be considered as falling below energy demand, can have catastrophic consequences on brain function (Rossen et al., 1943). Perhaps as a consequence of this increased energy demand the individual cells in the brain may have developed a degree of versatility with regard to which substrates they utilize under varying conditions (Ames, 2000).

In this review, we will describe experiments carried out in the central nervous system (CNS) that were designed to investigate the shuttling of lactate between glial cells and neurones, and the source of that lactate. The dogmatic view of all brain cells taking up glucose followed by complete oxidative phosphorylation has progressed to support the theory that there is co-operativity between glial cells and neurones imparted by the metabolic and physical compartmentalization between these two cells types.

## Glucose Metabolism

The role that glucose plays in supporting brain function is paramount and is accepted by all (Dienel, 2009). Corroborating information for this statement may be readily appreciated by realizing the following: (1) blood glucose is maintained within a narrow normoglycemic range (4 to 7.2 mM) via complex endocrine control mechanisms, strongly indicative that it is of paramount importance that blood glucose remains above a basal level, the obvious conclusion being that this basal level is above that required to ensure adequate delivery of glucose to the brain, (2) there are glucose sensitive neurones in the brain which function to induce compensatory mechanism that cause glucose to remain at a basal level, (3) the arterial to venous blood glucose difference is always negative i.e., the concentration of blood in the arterial delivery to the brain exceeds that of the venous drainage, consistent with extraction of glucose by the brain, (4) labeled glucose shows up as metabolites after introduction into the brain, (5) on introducing excess insulin into the systemic circulation the brain malfunctions, evidence that there is no alternative substrate present in sufficient concentrations in the systemic circulation to substitute for glucose, and (6) non-glucose substrates are converted into glucose in the liver and kidney via gluconeogenesis, implicating glucose as a preferred substrate that can be used by all cells (Frier et al., 2014). During starvation ketones can be act as alternate substrates but cannot fully substitute for glucose.

Studies into the distribution of glucose transporters have found these proteins present at the blood brain barrier (BBB) and also present on both neurones and glial cells, although the expression of the transporters is cell specific, with neurones tending to express Glut 3 and astrocytes Glut 1 (Choeiri et al., 2002; Thorens and Mueckler, 2010). This pattern suggests functional reasons for difference in expression, possibly due to the affinity and maximal rate of the transporter being suited to the particular requirements of individual cell types.

**FIGURE 1:**
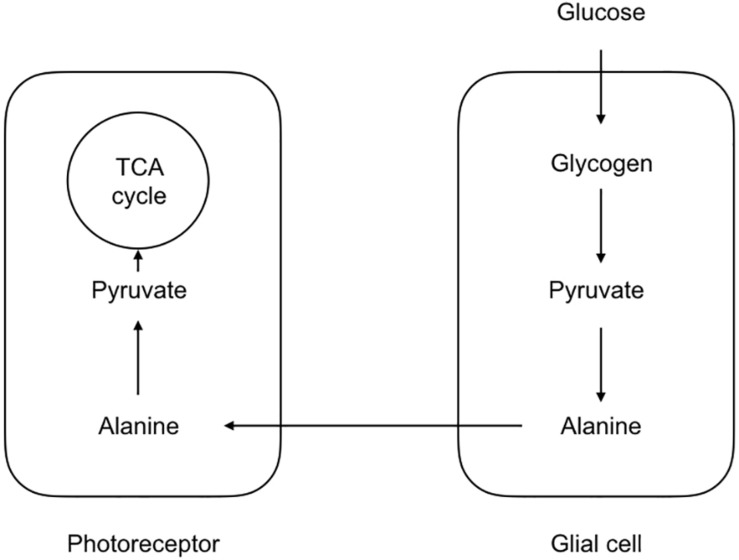
Morphological and metabolic compartmentalization in the honeybee retina. The glial components take up glucose, which is ultimately converted to alanine. The alanine is then shuttled to the photoreceptors where it is oxidatively metabolized. Such a scheme serves as a template for the lactate shuttling present in the mammalian CNS.

The first indication that not all cells take up glucose and fully metabolize it into carbon dioxide and water came for studies of brain metabolism where the ratio of oxygen uptake was compared to the glucose consumption. In the event of full oxidation of glucose the ratio would be 6, since 6 oxygen atoms would be required for the oxidation of the 6 carbon molecules in the glucose structure. However the value recorded varied at about 5.5 (Dienel, 2009). Although such recordings came from whole brain and gave no regional indications as to whether this discrepancy was due to one particular region of the brain or was a global phenomenon, it implied incomplete metabolism of glucose, which appears at odds with Rossen’s data, which clearly shows the brain’s avid requirement for energy, surely an indication that the brain would use all available energy supplied to it. However studies in rat have shown that while the brain does indeed extract glucose from the arterial delivery it also excretes the unused glucose into the venous drainage, suggesting that the brain takes up what glucose it requires for its immediate energy requirements and does not store the excess (McIlwain and Bachelard, 1985). Reasons for the disparity between the oxygen and glucose uptake ratio have lain at the center of a controversy that has engulfed the subject of brain energy metabolism for over the last 30 years. The first direct evidence for metabolic compartmentalization came from studies on the honeybee retina model (Tsacopoulos et al., 1994). Despite its non-mammalian lineage this is a very useful model with which to investigate metabolic compartmentalization since the neural and glial compartments are morphologically distinct and arranged in an organized and easily identifiable manner. In this model the glial elements take up the majority of the glucose, whereas the neural elements take up the majority of the oxygen. During periods of increased metabolic activity imposed by flashing light onto the retina, the glia glucose uptake increases, as does the neuronal oxygen consumption (Tsacopoulos et al., 1994). The model that emerges is shown in Figure 1 which may be considered the original lactate shuttle relevant to the CNS.

## Lactate Shutting in Mammalian Cns

**FIGURE 2:**
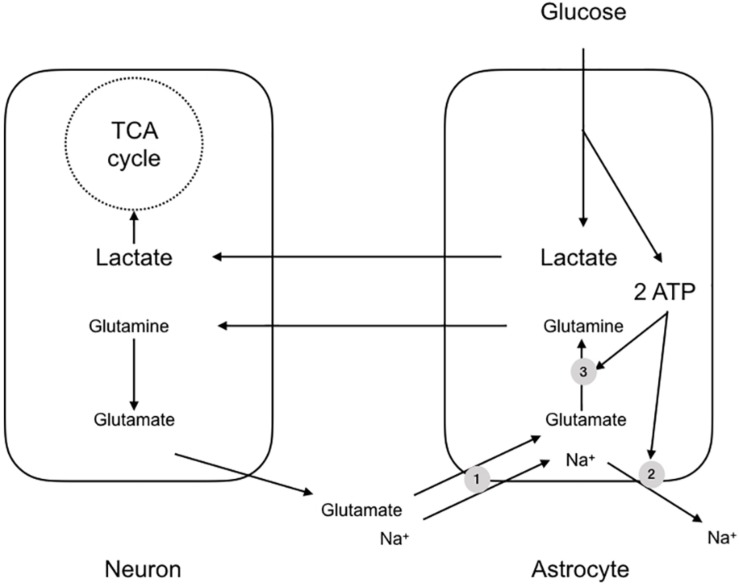
The astrocyte-neuron lactate shuttle hypothesis (ANLSH). In this scheme glutamate released at the synapse is taken up by Na^+^ coupled glutamate transporters (1). The glutamate is converted by glutaminase to glutamine, an ATP requiring reaction (3), with the glutamine transported to the presynaptic terminal as an inert compound that will not cause excitotoxicity. The Na^+^ that is transported with the glutamate is pumped out of the astrocyte by the energy requiring Na^+^ pump (2). The uptake of glutamate triggers glucose uptake into the astrocyte, which is glycolytically converted to lactate, producing two molecules of ATP. These ATP fuel the re-equilibration after glutamate uptake and the lactate is shuttled to the neurones for oxidative metabolism.

An almost contemporaneous report extended this scheme to apply to the mammalian brain, but with notable differences and additions. It was to prove one of the most contentious papers in the field of brain energy metabolism and the debate raging around the subject persists. The paper described experiments carried out using cultures of mouse cerebral neurones and astrocytes, and demonstrated a few key features, which were stoichiometrically robust (Pellerin and Magistretti, 1994). The background to the study was the realization that if metabolic signaling were to occur between neurones and astrocytes in the mammalian CNS, there must exist a signaling mechanism whereby the neurones signal to astrocytes their need for delivery of metabolic substrate. The signaling mechanism must have the following characteristics: (1) it must be related to the frequency of activity of the neurones i.e., the release of the compound must be related to the activity of the neurones, (2) it must be sensed by the astrocyte i.e., there must be mechanism(s) whereby the astrocyte senses the molecule, and (3) there must exist a system or process whereby the concentration of the molecule is decreased upon cessation of activity i.e., there must be an effective buffering system that reduces the concentration of the molecule after activity. In this study glutamate was shown to meet these three criteria (Pellerin and Magistretti, 1994). The release of synaptic glutamate is related to the frequency of action potentials, and it is buffered by astrocytes, causing its extracellular concentration to decrease rapidly after activity. Glutamate is taken up into astrocytes using the trans-membrane Na^+^ gradient (Sonnewald et al., 1997). The results of this increased glutamate uptake into astrocytes is an elevation in astrocytic Na^+^. In addition the glutamate that is taken up is converted into glutamine, a metabolically inert compound that can safely be released by the astrocyte for subsequent reuptake and cycling by the neurones without the risk of activation of glutamate receptors. However this reaction, catalyzed by glutamine synthase, requires ATP (Pellerin and Magistretti, 1994). Thus sequestering glutamate places a metabolic burden upon astrocytes due to glutamate-glutamine shuttling and extrusion of Na^+^ from the astrocyte via the ATP dependent Na^+^ pump. The uptake of glutamate coincides with an increased uptake of glucose from the media in a dose dependent manner (Pellerin and Magistretti, 1994) (Figure 2). Thus, the scheme that was proposed tied together these processes, whereby neuronal activation led to release of synaptic glutamate, which was taken up by astrocytes, in turn leading to an energy requiring processes to re-equilibrate ion gradients and shuttle glutamate. The glucose taken up by astrocytes was glycolytically metabolized to lactate. This offered the benefit of producing ATP for re-equilibrating the ion gradients and producing lactate, which could be shuttled to the neurones for oxidative metabolism (Pellerin and Magistretti, 1994).

There is insufficient space in this review to provide a detailed analysis of the ANLSH but there are numerous reviews, which either support or contest the concept (Korf, 2006; Mangia et al., 2009; DiNuzzo et al., 2010; Dienel, 2012; Pellerin and Magistretti, 2012). An alternate viewpoint is that the astrocytic glycogen is used exclusively for astrocytic metabolism, thereby preserving interstitial glucose for neuronal uptake (DiNuzzo et al., 2010). Allosteric regulation of glycogen by glucose phosphatases argues against the ANLSH. Glucose phosphates derived from astrocytic glycogen act as allosteric signals to reduce astrocytic uptake of glucose, thereby sparing interstitial glucose for neurons. Intracellular glucose-1-phosphate acts as a signal on the Na^+^/K^+^ pump to promote extracellular K^+^ buffering thereby controlling extracellular ion homeostasis (DiNuzzo, 2019).

## Brain Glycogen

What the proposal of the ANLSH did was bring into focus the manner in which neurones receive energy substrate. Before describing this in detail it would be useful first to describe the gross properties of whole body energy metabolism. The liver and skeletal muscles are the main depots of glycogen in the body (Stryer, 1995). The skeletal muscle glycogen is used as a localized energy source to fuel muscles, with the glycogen glycolytically metabolized to lactate, which is released from the muscle into the systemic circulation as a waste product (Dalsgaard et al., 2004). Liver glycogen is metabolized in response to falling systemic blood glucose levels and is released as glucose directly into the systemic circulation in order to maintain normoglycemic concentrations of blood glucose (Tymoczko et al., 2015). Since the brain is exquisitely sensitive to decreases in blood glucose (Frier et al., 2014), the role of the liver glycogen can be considered as maintaining an adequate delivery of glucose to the brain; the delivery of glucose to other organs is a consequence of the systemic circulation. There is glycogen present in the mammalian CNS but it is present in low concentrations relative to that in liver and skeletal muscle (Nelson et al., 1968). Measures of *ex vivo* or *in vivo* glycogen levels in rodents and humans are between 8 and 12 μmol g^–1^ and, assuming an astrocytic volume fraction of 15%, this gives a concentration of 50–80 μmol g^–1^, substantial compared to the ambient interstitial (glucose concentration). It must also be appreciated that except under conditions of severe systemic hypoglycemia glucose is always present in the interstitial fluid at 2–3 mM, thus glycogen acts to supplement a significant ambient glucose concentration. If the role of liver glycogen is primarily to fuel brain function, what then is the role of brain glycogen? Over the last 30 years a series of experiments have been carried which are beginning to reveal the physiological roles of brain glycogen.

The presence of glycogen in the mammalian brain has been known for decades (Cataldo and Broadwell, 1986). With the advent of electron microscopy and biochemical assay the presence and the content, respectively, of glycogen in all areas of the body was investigated. The presence of glycogen in the brain (Koizumi and Shiraishi, 1970a, b; Phelps, 1972; Koizumi, 1974) did not incite a detailed research program to uncover its role, principally as it occurred in such low concentrations relative to other areas of the body (Nelson et al., 1968). Elementary calculations demonstrated that the glycogen in the brain could only fuel brain function for a few minutes in the absence of glucose and thus its role was considered unimportant (Dienel, 2009). The fact that the glycogen was present in the astrocytes (Cataldo and Broadwell, 1986) and appeared to be localized to synaptic regions (Koizumi and Shiraishi, 1970a, b; Phelps, 1972; Koizumi, 1974) did not excite interest, and it was only when glial cells emerged from under the shadow of neurones and their importance in brain function was discovered that interest in brain glycogen was reawakened (Brown, 2004). Although some of the studies in the following descriptions are un-physiological (i.e., 0 mM glucose, 20 mM lacate in the aCSF, 100 Hz stimulus) they were important and warranted as they revealed basic properties and function of glycogen. The introduction of the cell culture technique in the 1980s, in which disparate cell types could cohabit in a petri dish bathed in a supportive medium, proved important. The co-culturing of neurones and glia led to the first important discovery, namely that neurones survive better in culture when astrocytes are also present (Whatley et al., 1981). This initial study did not reveal what aspect of the presence of astrocytes the neurones found essential for survival. This was not a trivial matter since the support afforded by astrocytes could be physical, where connections between cells are essential, or the release of some trophic factor from astrocytes that supported the neurones. A later culture study revealed that the essential component of astrocyte presence that supported neurones was the glycogen contained within the astrocytes (Swanson, 1992; Swanson and Choi, 1993). Astrocytes depleted of glycogen were not as successful at supporting neurones as astrocytes with a full complement of glycogen. It had been seen with preliminary electron microscopic studies that glycogen was located almost exclusively in astrocytes in adult mammalian brain (Cataldo and Broadwell, 1986). Only during development (Bloom and Fawcett, 1968) and pathological conditions (Vilchez et al., 2007) do neural elements express glycogen. This cellular location was intriguing for the following reasons. It was known that the cellular metabolic rates was higher in neurones than astrocytes (Dienel, 2009), and given the complex electrical activity that neurones display which underlies brain function, it would appear that the neuronal elements would require more energy than astrocytes. Consider that the maintenance of the resting membrane potential is a very energy dependent process, and that the firing of action and synaptic potentials disrupts this equilibrium, which must be reset at an energetic cost, this neural requirement for energy can be readily appreciated. Glycogen is a polymer of glucose in which dehydrated glucose molecules combine to from a large molecule with a molecular weight of up to 10^8^ (Champe and Harvey, 2008). Studies in culture have shown that astrocytes release lactate into the media (Dringen et al., 1995), which provided initial clues as to the mechanism whereby glycogen provides fuel. Glycogen is too large a molecule to be released and travel intercellularly. In addition it appears that glycogen’s role is not to provide energy substrate purely for astrocytes, thus the trafficking of lactate to neural elements makes sense, but does pose a few important questions. (1) How is the lactate transferred to the neural elements, (2) what is the stimulus or signaling mechanism by which neuronal elements signal to astrocytes of the energy requirements, (3) do astrocytes benefit from the presence of glycogen, and (4) is glycogen-derived lactate shuttled to neurones continuously or is it supplied on demand? An additional important question was what is the role of glucose uptake into neurones? Is this the dominant energy substrate used by neuronal elements, with lactate being a supplemental energy substrate taken up by neurones as and when it is required (Korf, 2006; DiNuzzo et al., 2010; Dienel, 2012; Dienel and Cruz, 2014)?

Initial studies by Pierre Magistretti’s group investigated the role of neurotransmitters and other compounds in regulating the glycogen content. They found that glycogen was indeed up- and down regulated by such compounds (Magistretti et al., 1993b, 1994; Sorg et al., 1995) and it was during the investigation of how glutamate affects glycogen content that the ANLSH scheme was uncovered (Pellerin and Magistretti, 1994). This labile property of glycogen makes functional sense since it means it can be up- and down regulated under variable physiological conditions, as yet unknown, to meet key demands.

## White Matter Glycogen Supports Cap Conduction

Studies by the Ransom laboratory used a different strategy to study glycogen, employing the rodent optic nerve as an experiential model of central white matter (Stys et al., 1991). The advantages of this model were that it lacked synapses and neurotransmitters, being composed of cable like myelinated axons, astrocytes and oligodendrocytes (Ransom et al., 1990). In addition axon conduction could be recorded in real time, thus by judicious addition or subtraction of energy substrates and other compounds in the aCSF that superfuses the tissue the role of key compounds could be investigated (Brown et al., 2001a, b). The initial study was carried out to see what the effect of removing glucose from the aCSF in the tissue was. This derived from investigations into the effects of ischemia on the tissue, which evolved into studies into the effects of the component parts of ischemia, namely anoxia and aglycemia, which were investigated separately. Anoxia (removal of oxygen) caused a rapid failure of the CAP after a few minutes (Stys et al., 1992). However removal of glucose in the presence of oxygen caused a delayed failure in the CAP, lasting up to 30 min in the absence of exogenously applied glucose (Ransom and Fern, 1997). This prolonged latency to CAP failure led to the assumption that the optic nerve must contain some form of energy reserve that sustains conduction for 30 min, but in addition, that the reserves are of limited size, and once they have been consumed CAP conduction fails (Ransom and Fern, 1997). The energy reserve proved to be glycogen, which is located in the astrocytes in the optic nerve (Wender et al., 2000). This discovery proved enlightening as it allowed for a multitude of experiments to be carried out in the model using different techniques to explore aspects of glycogen’s role.

**FIGURE 3:**
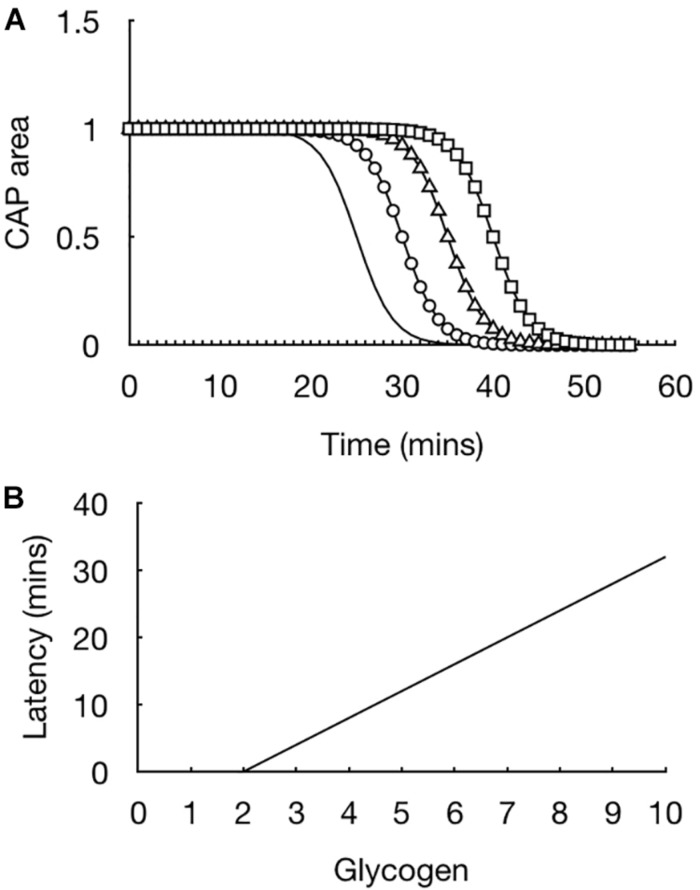
Glycogen content dictates latency to CAP failure in the MON model. **(A)** In MONs pre-incubated in 10 mM glucose (straight line), the CAP starts to fail at about 20 min after introducing 0 mM glucose aCSF, i.e., simulated aglycaemia, and falls rapidly to zero in the continued presence of aglycemia. In nerves pre-incubated for 2 h in increasing concentrations of glucose (circle – 15 mM, triangle – 20 mM, square – 30 mM), the latency to CAP failure increased in line with the glucose concentration. **(B)** There is a linear relationship between glycogen content (pmole μg protein^–1^) at the onset of aglycemia and latency to CAP failure.

The initial experiments were straightforward but provided extremely important information regarding how glycogen fuels the CAP. In light of evidence that the CAP was supported for 30 min prior to CAP failure the glycogen content in the tissue was measured in 10 min increments such that a picture could be built up of the dynamics of glycogen content in the face of aglycemia. At the onset of aglycemia the baseline glycogen content was measured. In parallel experiments the glycogen content was measured every 10 min and it was found that the glycogen content fell whereas the CAP was fully maintained (Wender et al., 2000). The glycogen reached its nadir at 30 min, which coincided with the point where the CAP fell (Wender et al., 2000). These data highlight the advantage of the rodent optic nerve as a model, as it allows two separate techniques to be applied to the tissue, whose results can be compared to provide a fuller picture as to glycogen’s role. The rate of fall of the glycogen was linear during the period of aglycemia. This suggested that if glycogen was unregulated prior to introducing aglycemia then the latency to CAP failure could be prolonged if glycogen was metabolized at the same steady rate. Glycogen can be unregulated by bathing the nerve in supra-physiological concentrations of glucose, thus incubating the nerve in 30 mM glucose for 2 h increases the glycogen content by a factor of about two compared to baseline levels, where the nerve is incubated in 10 mM glucose for 2 h (Wender et al., 2000). Exposing the optic nerve to aglycemia after increasing glycogen content did indeed lead to an increase in the latency to CAP failure. Incubating nerves in increasing concentrations of glucose such that a variety of glycogen levels were attained resulted in a linear relationship whereby the latency to CAP failure was determined by the glycogen content, i.e., increasing glycogen content in the nerve at the onset of aglycemia prolonged the latency to CAP failure. This was an important result for the following reason (Figure 3). It showed that glycogen was indeed metabolized to provide substrate to the tissue in the absence of exogenously applied energy substrate. In addition it demonstrated that the glycogen was metabolized at a steady rate with a constant energy demand by the tissue (Wender et al., 2000). These results are important for the diabetic community as they suggest that up regulating glycogen content may be an effective therapeutic strategy to stave off the effects of hypoglycemia (Frier et al., 2014). However, glycogen content would have to be appropriately regulated in order to avoid hypoglycemia awareness (Cryer, 2013).

Additional indirect evidence was provided to show that glycogen content determined the latency to CAP failure at the onset of aglycemia. Experiments were carried out where nerves were exposed to aglycaemia and the CAP recorded until it began to fall, an indication that glycogen was depleted. The CAP was allowed to recover to its baseline value and then aglycemia was introduced again, the idea being that the glycogen will not be replenished to its baseline level. The latency to CAP failure during this second period of aglycemia was shortened compared to the first period of aglycemia (Brown et al., 2003). The role of glycogen under more physiological conditions was investigated. In the presence of 2 mM glucose, which is considered to be hypoglycemic and a systemic concentration that is reached in type 1 diabetic patients who mismatch insulin delivery with prevailing glucose levels, the CAP is maintained for extended periods of time. However depleting glucose by imposing a period of aglycemia and then reintroducing 2 mM glucose led to CAP failure, indicating that on its own 2 mM glucose is not sufficient to support the CAP, but is supplemented by the breakdown of glycogen to provide supplemental energy substrate (Brown et al., 2003). Removing that source of glycogen-derived substrate leads to CAP failure, indicating that in type 1 diabetic patients, during periods of hypoglycemia, glycogen is broken down to provide supplemental substrate to support brain function. Given that this condition is hypoglycemia and not aglycemia, the glycogen content will not be as readily depleted thus the closer the blood glucose is to normoglycemic levels the longer it will take for glycogen to be depleted.

## Glycogen Derived Lactate Is Transported to Axons

**FIGURE 4:**
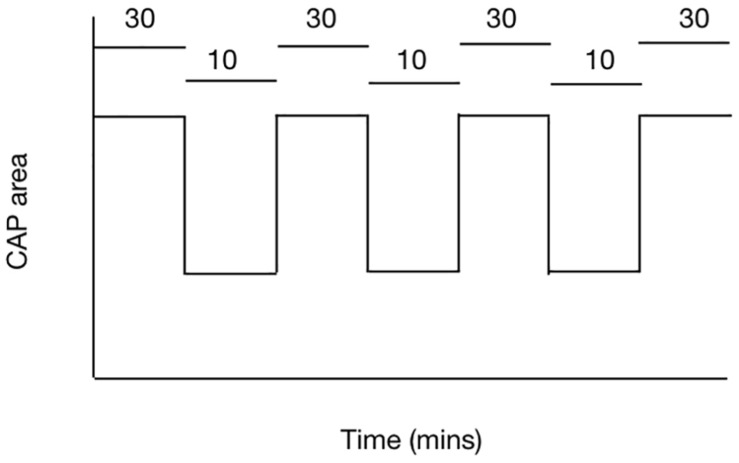
The ability of the MON to conduct CAPs is determined by the balance between tissue and energy demand and supply of substrate to the nerve. In MONs supplied with 10 mM glucose the imposition of 100 Hz stimulus causes the CAP area to fall. However replacing 10 mM glucose with 30 mM glucose in the aCSF restores the CAP to its full area. The horizontal bars indicate the glucose concentration present in the aCSF.

The balance between demand and supply can be investigated using the rodent optic nerve model. In nerves bathed with 10 mM glucose, imposing 100 Hz stimulus leads to a gradual fall in the CAP area, proof that the increased energy demand is not met by normoglycemic concentrations of glucose and the CAP cannot be fully supported under these conditions (Brown et al., 2003). However increasing the concentration of glucose to 30 mM leads to total CAP support for extended periods of time under 100 Hz stimulus (Brown et al., 2003) (Figure 4). These results suggest there is no absolute threshold for CAP conduction failure, but rather that as long as increases in demand are met by increases in supply then the CAP can be maintained.

The role that lactate plays in the above results was investigated. Firstly, if glycogen derived lactate is supporting the CAP, then introduction of exogenous lactate in the absence of glucose should support the CAP and this was shown to be the case (Brown et al., 2003). Such data imply that there is a means for lactate to enter axons (see later), and also indirectly for lactate to enter astrocytes, as astrocyte function is required for maintenance of the CAP, since astrocytes play important roles such as buffering K^+^ (Clausen, 1992). The use of the compound cinnemate, which blocks lactate uptake into axons, was strategically used to dissect the detail of lactate use. CIN, as well as D-lactate, must be used appropriately since they not only block membrane surface transporters but also block pyruvate uptake into mitochondria. In the presence of 2 mM glucose addition of 150 μM CIN caused a rapid CAP decrease, indicative of glycogen-derived lactate acting as a supplemental substrate in the presence of 2 mM glucose (Brown et al., 2003). However in the presence of 10 mM glucose CIN had no effect (Brown et al., 2003). This control experiment has far reaching consequences, as it suggests that not all glucose is shuttled via astrocytes, but that axons can directly take up glucose. In nerves exposed to 100 Hz when bathed in 10 mM glucose, the CAP failed suggesting that even in the presence of normoglycemic glucose additional energy substrate is required when the tissue energy demand increases. Such data are important as they show that glycogen has an important role under physiological conditions. The fact that glycogen supported CAP conduction in the absence of exogenously applied glucose probably has no value in ascribing a role to glycogen, since aglycaemia is an artificial experimental procedure, and hypoglycemia is an iatrogenic consequence of insulin therapy that did not exist prior to 1922 (Frier et al., 2014), thus cannot be glycogen’s main role. However supplying lactate to axons when tissue energy demand increases implies a novel role for brain glycogen and suggests it acts as an energy buffer rather than an energy reserve, whose role is not to single-handedly support conduction, but to act in concert with ambient levels of normoglycemic glucose. The implications of this are that such increases in demand can only be supported for finite periods of time, and once such events have occurred, there has to be a period of stability during which the glycogen can be replenished if it is to act as a viable buffer.

Further experiments investigated the transport of lactate from astrocytes to axons. In order for lactate to be used by axons there must be a means by which it leaves the astrocyte and is then taken up by the axon. This role is carried out by a specialized family of facilitated trans-membrane spanning transporters, which belong to the monocarboxylate family of transporters (MCTs). Immunohistochemical studies localized the MCT1 to the astrocyte and the MCT2 to the axon (Tekkok et al., 2005), which is consistent with the MCT1 releasing lactate from cells and the MCT2 taking lactate up into cells. The fact that CIN accelerates the latency to CAP failure during aglycemia is also supportive evidence that lactate is transferred from the astrocyte to the axon in the absence of exogenously applied glucose (Brown et al., 2003). Further studies which exploited the ability of imposition of 100 Hz stimulus to increase tissue energy demand were employed to study the role of lactate. When 100 Hz stimulus was imposed in optic nerves superfused with 10 mM glucose after a period of 4 min the CAP was maintained at its baseline level (Brown et al., 2003). However the CAP fell when either the glycogen metabolism blocker isofagomine was added 20 min prior to the test stimulus (Brown et al., 2005), or if 150 μM CIN or 20 mM D-lactate, the non-metabolizable isomer of L-lactate that is transported at the MCT but is not metabolized, were added (Tekkok et al., 2005). These results add credence to the hypothesis that lactate is transferred to the axons under physiological conditions (albeit extremely high frequency firing) when superfused with normoglycemic concentrations of glucose. Thus optic nerve axons clearly show a degree of versatility depending upon immediate energy expenditure and substrate requirement. In the presence of normoglycemic glucose (10 mM) blockade of lactate uptake into the axon by addition of CIN has no effect on the CAP (Brown et al., 2005), indicating that under such conditions there is no absolute requirement for lactate uptake into the axon. Whether this occurs under control conditions is not known, and it may well be that under such baseline conditions the axons take up both glucose and lactate, but when the route of lactate uptake is blocked the axons simply increases its uptake of glucose to make up for the shortfall in lactate delivery. The experiments, which showed CAP failure when lactate uptake was blocked in optic nerves perfused with 2 mM glucose (Brown et al., 2005), demonstrates that only when sufficient glucose is present can the axon continue to conduct the CAP when lactate uptake is blocked. This is supported by data, which shows that 10 mM glucose can support the CAP under 100 Hz for a short period of time, but that the CAP fails if lactate uptake into axons is blocked (Tekkok et al., 2005). However extended periods of 100 Hz stimulus leads to CAP failure in optic nerves perfused with 10 mM glucose. However replacing the 10 mM glucose with 30 mM glucose restores the CAP to it baseline size. Under these conditions in 10 mM glucose there is insufficient energy substrate intake, both in the form of glucose and lactate to support the CAP under 100 Hz but given sufficient glucose the CAP is restored (Brown et al., 2003). This versatility is interesting as it supposes that axons are flexible in the substrate they can use – either glucose or lactate depending upon both availability and the nature of demand.

## Dynamics of Lactate Flux

Thus the presumed release of lactate from astrocytes is clearly indicative of their ability to survive on the proceeds of glycolysis. The use of lactate biosensors affords the opportunity to determine whether lactate is released for astrocytes and what it’s dynamics are under varying experimental conditions. A steady concentration of between 50 and 300 μM lactate is measured when lactate biosensors are pressed against the *ex vivo* optic nerve in a chamber superfused with aCSF containing 10 mM glucose but lacking any lactate (Yang et al., 2014). This implies that the tissue continually releases a constant amount of lactate into the interstitial space under baseline conditions. However it must be appreciated that these sensors are recording the lactate outside the nerve, so the interstitial concentration is likely to be considerable higher. In addition, given the lack of lactate in the aCSF, there is a very large concentration gradient forcing lactate out of the tissue, which differs from the *in vivo* situation where there is a constant lactate presence in the interstitium. A steady recording of lactate implies a constant efflux from the tissue as we are in effect recording a net flux, rather than a static concentration, since the nerve is continually superfused with aCSF at a rate of 2 ml min^–1^. These data do indeed imply that astrocytes release lactate that is available to the axons for uptake (Yang et al., 2014), but it must be appreciated that under such circumstances there is a large gradient for lactate to move from the interstitium out of the nerve as there is no lactate in the aCSF superfusing the nerve.

**FIGURE 5:**
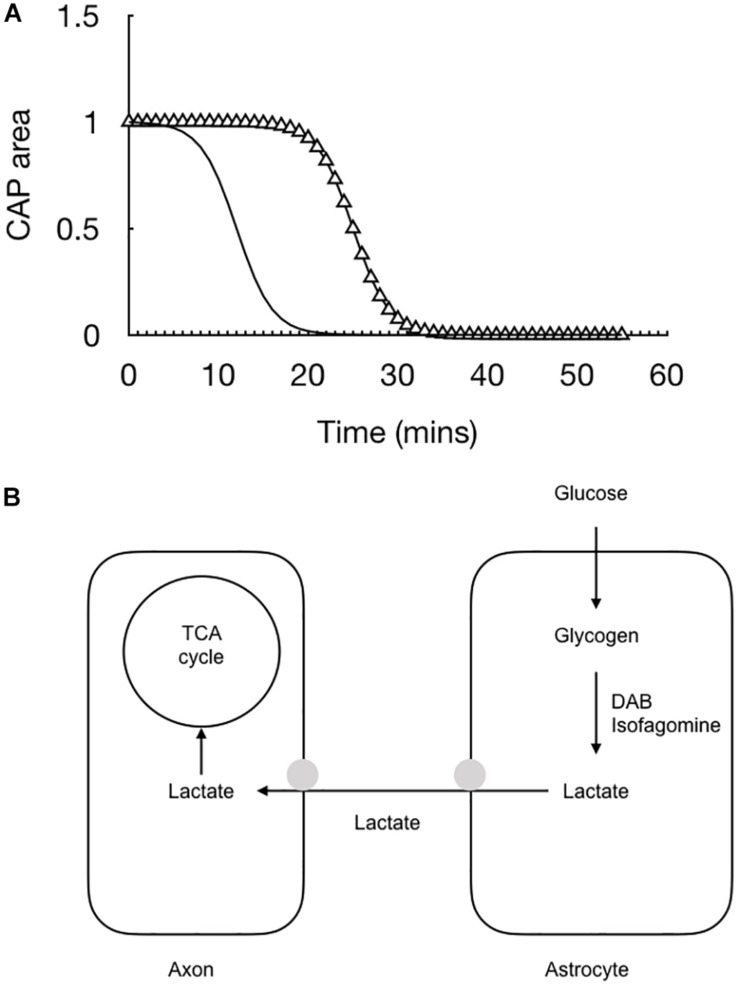
The combination of recording the stimulus evoked CAP (triangle) with real time recordings of lactate (black line) allow for a fuller picture of the cellular interactions to emerge. **(A)** At the onset of aglycemia the lactate falls almost immediately followed by the CAP. **(B)** Glucose is taken up by astrocytes and either stored as glycogen or directly processed glycolytically to lactate. The lactate is then transported to the axons for oxidative metabolism. Removing glucose from the aCSF or inhibiting glycogen metabolism with DAB or isofagomine causes the lactate to rapidly fall to zero followed by the CAP.

The lactate falls rapidly to zero when glucose is removed from the aCSF – indeed it’s decline precedes by some 10 min the failure of the CAP (Yang et al., 2014). We assume from this that in the absence of exogenously applied glucose the axons switch to exclusively metabolizing lactate derived from astrocytic glycogen and the rapid fall is indicative of axons taking up all available lactate that is shuttled into the interstitial space and hence no lactate is recorded by the sensor (Figure 5).

The conclusion from this optic nerve data is that astrocytes contain glycogen and release glycogen derived lactate into the interstitial space via the MCT1, from where it is taken up into axons via the MCT2 transporter. In addition to this lactate release under conditions of aglycemia, where the latency to CAP failure can be accelerated by blocking lactate uptake or inhibiting glycogen metabolism, lactate is also released tonically under baseline conditions. The reason for this is not known but clearly shows that astrocytes do not oxidatively metabolize all the glucose they take up. The putative role for this lactate is open to debate. It may be released from the astrocyte as a form of energy substrate that is immediately available to axons at the onset of increased activity. This is supported by data from *in vivo* recordings of cat cortex in which lactate sensors inserted into the cortex of a live cat temporarily falls at the onset of experimentally induced activity. The temporary dip is followed by a rise in lactate in response to the continuous stimulus (Hu and Wilson, 1997). The initial dip is assumed to be due to the onset of activity requiring an immediate increase in delivery of substrate to the neurones. The lactate in the interstitial space meets this criterion. The latter increase in lactate is presumably due to the mechanism whereby neurones signal to astrocytes of their increased energy requirements, and the astrocytes respond by releasing lactate. It is interesting to note the unexpected results that axons in the optic nerve can survive in the absence of oxygen (Baltan Tekkök et al., 2003), which confounds expectations of an absolute requirement for oxygen of central nervous system neurones. Either the removal of oxygen or the addition of cyanide, which inhibits the complexes on the cristae of the mitochondria where the election transport chain occurs, leads to a fall in the CAP by 70%, but the important and unexpected result is that many axons survive and continue to fire under these conditions (Baltan Tekkök et al., 2003). Equivalent experiments in the hippocampus or the cortex lead to a rapid and total loss of neuronal firing (Tian and Baker, 2000). These data suggest versatility in axon energy requirements that support the glycogen data. When the optic nerve is supplied exclusively with lactate the CAP can be maintained for a period of several hours (Brown et al., 2003) indicating that it can survive exclusively on the proceeds of oxidative metabolism and does not have an absolute requirement for glycolysis. These data tend to support at least a partial compartmentalization of metabolism, where astrocytes tend to be glycolytic and release lactate, whereas a large proportion of axons tend to be oxidative, but a significant subset of axons can survive for extended periods of time on the proceeds of glycolysis (Baltan Tekkök et al., 2003). That all axons are supported by lactate does not support a scenario where a distinct proportion of axons are exclusively glycolytic (i.e., all axons contain mitochondria), but rather points toward versatility dependent upon ambient conditions and levels of glucose and lactate.

As well as acting as a transportable metabolic conduit, lactate may also act independently as a signaling molecule. Lactate inhibits neuronal activity through GPCR and HCAR1, thereby promoting glucose diffusion to regions of high activity and low concentration (Barros, 2013). Lactate is also proposed to enhance memory by acting to enhance NMDA mediated currents, which induces expression of the genes *Arc*, *Egr 1* and *Bdnf* via the redox state of the neuron (Magistretti and Allaman, 2018), a mechanism also proposed by which lactate acts as a more general signaling molecule (Mosienko et al., 2015).

## Glycogen in Gray Matter

We can now focus on the result from other areas of the brain, notable the gray matter cortex and hippocampus, to try to reach a consensus as to the roles of glycolysis and oxidative metabolism. In the brain the cortex and the hippocampal slice can be supported by lactate but its conduction does tend to fall (Bachelard et al., 1984; Cater et al., 2001, 2003), which may either be due to run down of the tissue or a suggestion of incomplete support. A difficulty in looking at gray matter is that the interactions and diversity of cells exceeds that of the white matter optic nerve, thus results can be confusing. It is tempting to rely on such reduced simpler systems as tissue culture, but the translatability of these to the *in vivo* brain are not entirely convincing. This is especially true where under *in vivo* conditions there is a large intracellular compartment and a small interstitial space, whereas under tissue culture conditions the extracellular volume i.e., the media, is infinitely large compared to the intracellular compartments. It is particularly difficult to assess the degree of cell-to-cell communication under tissue culture conditions. Thus the ANLSH data proposed by Pellerin and Magistretti, who derived their hypothesis from tissue culture experiments, must be viewed under these conditions. Unfortunately, there is no system that is as simple or compartmentalized as the honeybee retina, so these types of experiment tend to be compromises at best, with conjecture and implication replacing convincing experimental evidence.

The advance of technology has improved our knowledge of such cellular transactions. FRET sensors can be manufactured to respond to individual compounds (Bittner et al., 2010). The lactate sensor has been used to investigate the release of lactate from cultured astrocytes in combination with patch clamp recordings. These data revealed a lactate channel that was present, which had an amplitude of 37 pS, and was activated by cell depolarization, which could be achieved by elevating extracellular [K^+^] (Sotelo-Hitschfeld et al., 2015). Within the astrocytes there was a pool of lactate estimated at about 1.3 mM, which could be released very quickly in response to local neuronal activation. This channel is distinct from the MCT and therefore does not rely on the trans-membrane concentrations of lactate and H^+^, with lactate able to be released against its concentration gradient (Sotelo-Hitschfeld et al., 2015). This is an intriguing result as it adds to the mechanisms by which lactate can be released from astrocytes.

In order for astrocytic glycogen to benefit neurones during increased neuronal activity there must exist a signaling molecule that is released by neurones during increased activity that can be sensed by astrocytes in an activity dependent manner. Although initial interest focused on glutamate (Pellerin and Magistretti, 1994) it now seems more likely that interstitial K^+^ is the most likely candidate. Whereas glutamate is restricted to particular areas of the brain, K^+^ is released by all axons as a result of increased activity (Hodgkin and Huxley, 1947, 1953), so is ideally positioned to be a universal signaling molecule. In hippocampal slice experimentally induced elevations in [K]o within the normal physiological range (3–12 mM) increased glycogenolysis and ultimately release of lactate into the interstitial space. The mechanism underlying this process is K^+^ induced stimulation of the Na^+^ coupled HCO_3_ transporter that results in increases in HCO_3_ uptake and intracellular alkalization. This activates soluble adenylyl cyclase, which increases cAMP levels promoting glycogen breakdown (Choi et al., 2012). Whether this mechanism applies to all areas of the nervous system areas is unknown, but experiments where genetically encoded FRET nanosensors for ATP were used, demonstrated increased intracellular ATP levels in astrocytes whereas intracellular neuronal ATP levels remain constant in response to increased extracellular K^+^ (Lerchundi et al., 2019). The increase in extracellular K^+^ that results from neuronal activity triggers astrocytic glycolysis to supply sufficient lactate for neuronal use as an energy substrate and intercellular signal in the hippocampus (Ruminot et al., 2019).

## Glycogen and Memory

To date the most important functional role ascribed to lactate has been that of facilitating learning and memory. Initial studies in 1 day old chick demonstrated that exposure to an experimental paradigm designed to invoke learning resulted in a decrease in glycogen, and a temporally correlated elevation in interstitial glutamate, suggesting that learning promotes glycogen metabolism to produce glutamate, an anaplerotic reaction, that is subsequently vital to the learning protocol. The memory consolidation was attenuated when glycolysis was inhibited via the used of iodoacetate or 2-deoxyglucose, effects that could be circumvented by the application of acetate, which is produced by astrocytes. Blocking glycogen metabolism via the use of DAB, an inhibitor of glycogen phosphorylase, attenuated the learning process, which could again be circumvented by the addition of glutamine, aspartate or acetate. The overall scheme that emerged was one in which the memory storage in the chick involves three distinct processes, (1) short term recall, (2) intermediate memory, and (3) memory consolidation into long-term memory (O’Dowd et al., 1994; Hertz et al., 1996, 2003; Gibbs et al., 2006a, b). The discovery that there is a metabolic component to memory consolidation is an extremely important one that adds additional complexity to a topic whose mechanism has provided considerable debate since the initial studies in rabbit that correlated the experimental paradigm of long term potentiation with the retention of memories (Bliss and Lomo, 1973).

**FIGURE 6:**
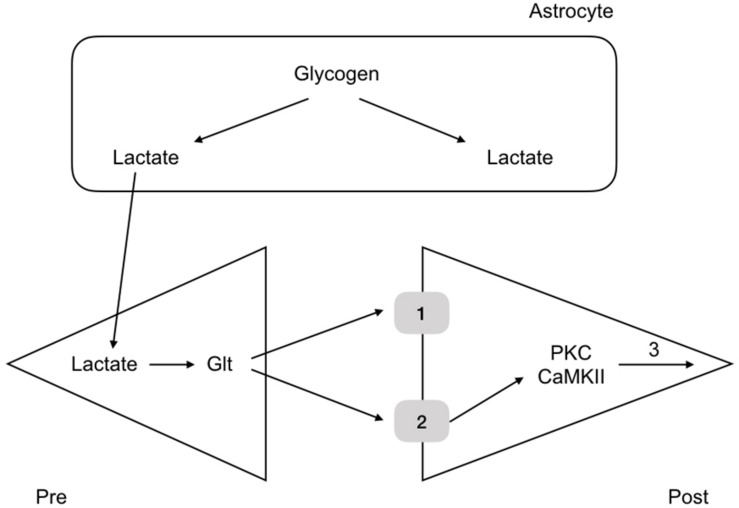
Glycogen metabolism underlies memory consolidation. Glycogen, located in astrocytes in the hippocampus, is metabolized to lactate, which is transported to the pre and postsynaptic terminals, where it fulfils separate roles, being converted to glutamate in the presynaptic terminal, and aiding consolidation in the postsynaptic terminal.

More recent experiments in rat have revealed the mechanism by which glycogen regulates memory. Rats were housed in one chamber and on opening a door, the rats were allowed access to a second chamber. The time it took the rats, naturally inquisitive animals, to enter the room was recorded. When rats had entered the second chamber they were given a foot shock, which served as an aversive stimulus. The rats were then placed back in the first chamber and the time taken to enter the second chamber recorded. The time taken after the conditioning foot shock was an indication of memory, an increase in time an indication that the rat had remembered the aversive shock. Rats were tested over the course of 7 days and the results showed that the latency to enter the second chamber increased after the conditioning aversive stimuli (Suzuki et al., 2011). However administration of DAB, a glycogen phosphorylase inhibitor, 15 min prior to the shock attenuated the latency, indicating that the memory was not as robust if glycogen metabolism was inhibited. Introducing lactate into the interstitial space via a dialysis probe subsequently increased the latency to entering the room, an indication that it is not the glycogen as such, but the lactate derived from glycogen, and presumably delivered to the neurones, that is the decisive factor in the memory process (Suzuki et al., 2011). A variety of other manipulations of lactate transfer involving down regulating the MCTs attenuated the retention time of the memory (Suzuki et al., 2011). The conclusion from these experiments is that glycogen derived lactate transferred from the astrocytes to the neurones is a requirement for laying down memory (Figure 6). These data were supported by studies in which the glycogen synthase enzyme was knocked out and there was impairment in the acquisition process of learning (Duran et al., 2013). There is evidence that up-regulation of key astrocyte-neuron metabolic coupling genes is important for memory following learning (Tadi et al., 2015).

Recently it has been shown that neuronal activity regulates many astrocytic genes, in particular up-regulation of genes associated with glycolysis and lactate, but not oxidative metabolism. Therefore neurones clearly influence the astrocytic transcriptome particularly genes of the lactate shuttle (Hasel et al., 2017). Localization of glycogen in the hippocampus using antibodies and microwave fixation provides evidence for glycogen rich or poor astrocytes, and the role of Na^+^ and K^+^ as regulators of glycogen content (Hirase et al., 2019).

## Glycogen and Neurological Function

The dogmatic view of cellular expression of glycogen in the brain is that it is exclusively loctated in astrocytes in adults, and is only expressed in neurones during development (Bloom and Fawcett, 1968; Magistretti et al., 1993a) or as a result of pathology (Vilchez et al., 2007). However with age neurons show an increased ability to metabolize glycogen and perform glycolysis, thereby demonstrating a shift from dependence to independence on astrocytic lactate (Drulis-Fajdasz et al., 2018). The reasons for this are unknown but may well have significance to an aging population.

There is evidence that decreased glycogen levels in the brain can cause neurological disruption. Depleted glycogen levels or sleep deprivation promotes cortical spreading depression due to disrupted K^+^ regulation by astrocytes (Kilic et al., 2018), and altered astrocyte morphology and decreased glycogen content induced as a result of chronic stress (Zhao et al., 2017).

Peripheral L-lactate administration has an antidepressant like effect which is linked to increased hippocampal lactate levels and regulation of a variety of signaling molecules and genes associated with serotonin signaling, astrocyte function, inflammation, NOS production, neurogenesis and synaptic function (Carrard et al., 2018).

Astrocytic glycogen metabolism is shown to be fundamental to many physiological processes with many diseases associated with abnormal glycogen metabolism, learning and memory, Alzheimer’s disease, epilepsy, sleep and diabetes (Bak et al., 2018).

## Conclusion

The presence of glycogen in the brain has been recognized for decades, but it is only recently that it has been assigned functional roles. It occurs at too low a concentration to be considered a conventional energy reserve, and must be considered as a buffer to supply energy substrate in the form of glycogen derived lactate, which is transported intercellularly to fuel neurones during periods of increased energy demand, such as occurs during the process of learning and memory.

## Author Contributions

AB was responsible for the conception of the review. WH and LR contributed to the review and approved the final version of the manuscript.

## Conflict of Interest

The authors declare that the research was conducted in the absence of any commercial or financial relationships that could be construed as a potential conflict of interest.

## References

[B1] Ames A. (2000). 3rd, CNS energy metabolism as related to function. *Brain Res. Rev.* 34 42–68. 10.1016/s0165-0173(00)00038-2 11086186

[B2] Bachelard H. S. Cox D. W. Drower J. (1984). Sensitivity of guinea-pig hippocampal granule cell field potentials to hexoses in vitro: an effect on cell excitability? *J. Physiol.* 352 91–102. 10.1113/jphysiol.1984.sp015279 6747907 PMC1193199

[B3] Bak L. K. Walls A. B. Schousboe A. Waagepetersen H. S. (2018). Astrocytic glycogen metabolism in the healthy and diseased brain. *J. Biol. Chem.* 293 7108–7116. 10.1074/jbc.R117.803239 29572349 PMC5950001

[B4] Baltan Tekkök S. Brown A. M. Ransom B. R. (2003). Axon function persists during anoxia in mammalian white matter. *J. Cereb. Blood Flow Metab.* 23 1340–1348. 14600441 10.1097/01.WCB.0000091763.61714.B7

[B5] Barros L. F. (2013). Metabolic signaling by lactate in the brain. *Trends Neurosci.* 36 396–404. 10.1016/j.tins.2013.04.002 23639382

[B6] Bittner C. X. Loaiza A. Ruminot I. Larenas V. Sotelo-Hitschfeld T. Gutierrez R. (2010). High resolution measurement of the glycolytic rate. *Front. Neuroenerg.* 2:26. 10.3389/fnene.2010.00026 20890447 PMC2947927

[B7] Bliss T. V. Lomo T. (1973). Long-lasting potentiation of synaptic transmission in the dentate area of the anaesthetized rabbit following stimulation of the perforant path. *J. Physiol.* 232 331–356. 10.1113/jphysiol.1973.sp010273 4727084 PMC1350458

[B8] Bloom W. Fawcett D. W. (1968). *A Textbook of Histology.* London: W. B Co.

[B9] Boron W. F. Boulpaep E. L. (2009). *Medical Physiology.* Amsterdam: Elsevier.

[B10] Brown A. M. (2004). Brain glycogen re-awakened. *J. Neurochem.* 89 537–552. 10.1111/j.1471-4159.2004.02421.x 15086511

[B11] Brown A. M. Sickmann H. M. Fosgerau K. Lund T. M. Schousboe A. Waagepetersen H. S. (2005). Astrocyte glycogen metabolism is required for neural activity during aglycemia or intense stimulation in mouse white matter. *J. Neurosci. Res.* 79 74–80. 10.1002/jnr.20335 15578727

[B12] Brown A. M. Tekkok S. B. Ransom B. R. (2003). Glycogen regulation and functional role in mouse white matter. *J. Physiol.* 549 501–512. 10.1113/jphysiol.2003.042416 12679378 PMC2342948

[B13] Brown A. M. Wender R. Ransom B. R. (2001a). Ionic mechanisms of aglycemic axon injury in mammalian central white matter. *J. Cereb. Blood Flow Metab.* 21 385–395. 10.1097/00004647-200104000-00007 11323524

[B14] Brown A. M. Wender R. Ransom B. R. (2001b). Metabolic substrates other than glucose support axon function in central white matter. *J. Neurosci. Res.* 66 839–843. 10.1002/jnr.10081 11746409

[B15] Carrard A. Elsayed M. Margineanu M. Boury-Jamot B. Fragniere L. Meylan E. M. (2018). Peripheral administration of lactate produces antidepressant-like effects. *Mol. Psychiatry* 23 392–399. 10.1038/mp.2016.179 27752076 PMC5794893

[B16] Cataldo A. M. Broadwell R. D. (1986). Cytochemical identification of cerebral glycogen and glucose-6-phosphatase activity under normal and experimental conditions. I. Neurons and glia. *J. Elec. Micro. Techol.* 3 413–437. 10.1002/jemt.1060030406 3018177

[B17] Cater H. L. Benham C. D. Sundstrom L. E. (2001). Neuroprotective role of monocarboxylate transport during glucose deprivation in slice cultures of rat hippocampus. *J. Physiol.* 531 459–466. 10.1111/j.1469-7793.2001.0459i.x 11230518 PMC2278461

[B18] Cater H. L. Chandratheva A. Benham C. D. Morrison B. Sundstrom L. E. (2003). Lactate and glucose as energy substrates during, and after, oxygen deprivation in rat hippocampal acute and cultured slices. *J. Neurochem.* 87 1381–1390. 10.1046/j.1471-4159.2003.02100.x 14713294

[B19] Champe P. C. Harvey R. A. (2008). *Biochemistry.* Baltimore, MD: Lippincott Williams & Wilkins.

[B20] Choeiri C. Staines W. Messier C. (2002). Immunohistochemical localization and quantification of glucose transporters in the mouse brain. *Neuroscience* 111 19–34. 10.1016/s0306-4522(01)00619-4 11955709

[B21] Choi H. B. Gordon G. R. Zhou N. Tai C. Rungta R. L. Martinez J. (2012). Metabolic communication between astrocytes and neurons via bicarbonate-responsive soluble adenylyl cyclase. *Neuron* 75 1094–1104. 10.1016/j.neuron.2012.08.032 22998876 PMC3630998

[B22] Clausen T. (1992). Potassium and sodium transport and pH regulation. *Can. J. Physiol. Pharmacol.* 70 S219–S222. 1295672 10.1139/y92-265

[B23] Cryer P. E. (2013). Mechanisms of hypoglycemia-associated autonomic failure in diabetes. *N. Engl. J. Med.* 369 362–372. 23883381 10.1056/NEJMra1215228

[B24] Dalsgaard M. K. Ogoh S. Dawson E. A. Yoshiga C. C. Quistorff B. Secher N. H. (2004). Cerebral carbohydrate cost of physical exertion in humans. *Am. J. Physiol. Regul. Integr. Comp. Physiol.* 287 R534–R540. 15155282 10.1152/ajpregu.00256.2004

[B25] Davies P. (1998). *The Fifth Miracle.* London: Penguin press.

[B26] Dienel G. A. (2009). “Energy metabolism in the brain,” in *From Molecules to Networks: An Introduction to Cellular and Molecular Neuroscience*, eds Byrne J. H. Roberts J. L., (Cambridge, MA: Academic Press), 49–110.

[B27] Dienel G. A. (2012). Brain lactate metabolism: the discoveries and the controversies. *J. Cereb. Blood Flow Metab.* 32 1107–1138. 10.1038/jcbfm.2011.175 22186669 PMC3390802

[B28] Dienel G. A. Cruz N. F. (2014). Contributions of glycogen to astrocytic energetics during brain activation. *Metab. Brain Dis.* 30 281–298. 10.1007/s11011-014-9493-8 24515302 PMC4130810

[B29] DiNuzzo M. (2019). How glycogen sustains brain function: a plausible allosteric signaling pathway mediated by glucose phosphates. *J. Cereb. Blood Flow Metab.* 39 1452–1459. 10.1177/0271678X19856713 31208240 PMC6681540

[B30] DiNuzzo M. Mangia S. Maraviglia B. Giove F. (2010). Glycogenolysis in astrocytes supports blood-borne glucose channeling not glycogen-derived lactate shuttling to neurons: evidence from mathematical modeling. *J. Cereb. Blood Flow Metab.* 30 1895–1904. 10.1038/jcbfm.2010.151 20827264 PMC3002884

[B31] Djokic T. Van Kranendonk M. J. Campbell K. A. Walter M. R. Ward C. R. (2017). Earliest signs of life on land preserved in ca. 3.5 Ga hot spring deposits. *Nat. Commun.* 8:15263. 10.1038/ncomms15263 28486437 PMC5436104

[B32] Dringen R. Peters H. Wiesinger H. Hamprecht B. (1995). Lactate transport in cultured glial cells. *Dev. Neurosci.* 17 63–69. 10.1159/000111275 7555739

[B33] Drulis-Fajdasz D. Gizak A. Wojtowicz T. Wisniewski J. R. Rakus D. (2018). Aging-associated changes in hippocampal glycogen metabolism in mice. Evidence for and against astrocyte-to-neuron lactate shuttle. *Glia* 66 1481–1495. 10.1002/glia.23319 29493012 PMC6001795

[B34] Duran J. Saez I. Gruart A. Guinovart J. J. Delgado-Garcia J. M. (2013). Impairment in long-term memory formation and learning-dependent synaptic plasticity in mice lacking glycogen synthase in the brain. *J. Cereb. Blood Flow Metab.* 33 550–556. 10.1038/jcbfm.2012.200 23281428 PMC3618391

[B35] Frier B. M. Heller S. R. McCrimmon R. J. (2014). *Hypoglycaemia in Clinical Diabetes.* Chichester: Wiley and Sons.

[B36] Gale J. (2009). *Astrobiology of Earth.* Oxford: Oxford University Press.

[B37] Gibbs M. E. Anderson D. G. Hertz L. (2006a). Inhibition of glycogenolysis in astrocytes interrupts memory consolidation in young chickens. *Glia* 54 214–222. 10.1002/glia.20377 16819764

[B38] Gibbs M. E. O’Dowd B. S. Hertz E. Hertz L. (2006b). Astrocytic energy metabolism consolidates memory in young chicks. *Neuroscience* 141 9–13. 10.1016/j.neuroscience.2006.04.038 16750889

[B39] Harris J. J. Attwell D. (2012). The energetics of CNS white matter. *J. Neurosci.* 32 356–371. 10.1523/JNEUROSCI.3430-11.2012 22219296 PMC3272449

[B40] Hasel P. Dando O. Jiwaji Z. Baxter P. Todd A. C. Heron S. (2017). Neurons and neuronal activity control gene expression in astrocytes to regulate their development and metabolism. *Nat. Commun.* 8:15132. 10.1038/ncomms15132 PMC5418577 28462931

[B41] Hertz L. Gibbs M. E. O’Dowd B. S. Sedman G. L. Robinson S. R. Sykova E. (1996). Astrocyte-neuron interaction during one-trial aversive learning in the neonate chick. *Neurosci. Biobehav. Rev.* 20 537–551. 8880738 10.1016/0149-7634(95)00020-8

[B42] Hertz L. O’Dowd B. S. Ng K. T. Gibbs M. E. (2003). Reciprocal changes in forebrain contents of glycogen and of glutamate/glutamine during early memory consolidation in the day-old chick. *Brain Res.* 994 226–233. 10.1016/j.brainres.2003.09.044 14642648

[B43] Hirase H. Akther S. Wang X. Oe Y. (2019). Glycogen distribution in mouse hippocampus. *J. Neurosci. Res.* 97 923–932. 10.1002/jnr.24386 30675919

[B44] Hodgkin A. L. Huxley A. F. (1947). Potassium leakage from an active nerve fibre. *J. Physiol.* 106 341–367. 10.1113/jphysiol.1947.sp004216 16991765 PMC1393796

[B45] Hodgkin A. L. Huxley A. F. (1953). Movement of radioactive potassium and membrane current in a giant axon. *J. Physiol.* 121 403–414. 10.1113/jphysiol.1953.sp004954 13085343 PMC1366083

[B46] Hu Y. Wilson G. S. (1997). A temporary local energy pool coupled to neuronal activity: fluctuations of extracellular lactate levels in rat brain monitored with rapid-response enzyme-based sensor. *J. Neurochem.* 69 1484–1490. 10.1046/j.1471-4159.1997.69041484.x 9326277

[B47] Kilic K. Karatas H. Donmez-Demir B. Eren-Kocak E. Gursoy-Ozdemir Y. Can A. (2018). Inadequate brain glycogen or sleep increases spreading depression susceptibility. *Ann. Neurol.* 83 61–73. 10.1002/ana.25122 29244233

[B48] Koizumi J. (1974). Glycogen in the central nervous system. *Prog. Histochem. Cytochem.* 6 1–37. 10.1016/s0079-6336(74)80003-3 4218335

[B49] Koizumi J. Shiraishi H. (1970a). Glycogen accumulation in dendrites of the rabbit pallidum following trifluoperazine administration. *Exp. Brain Res.* 11 387–391. 5496937 10.1007/BF00237912

[B50] Koizumi J. Shiraishi H. (1970b). Ultrastructural appearance of glycogen in the hypothalamus of the rabbit following chlorpromazine administration. *Exp. Brain Res.* 10 276–282. 5441445 10.1007/BF00235051

[B51] Korf J. (2006). Is brain lactate metabolized immediately after neuronal activity through the oxidative pathway? *J. Cereb. Blood Flow Metab.* 26 1584–1586. 10.1038/sj.jcbfm.9600321 16639423

[B52] Lane N. (2015). *The Vital Question.* London: Profile Publishing.

[B53] Lerchundi R. Kafitz K. W. Winkler U. Farfers M. Hirrlinger J. Rose C. R. (2019). FRET-based imaging of intracellular ATP in organotypic brain slices. *J. Neurosci. Res.* 97 933–945. 10.1002/jnr.24361 30506574

[B54] Magistretti P. J. Allaman I. (2018). Lactate in the brain: from metabolic end-product to signalling molecule. *Nat. Rev. Neurosci.* 19 235–249. 10.1038/nrn.2018.19 29515192

[B55] Magistretti P. J. Sorg O. Martin J.-L. (1993a). “Regulation of glycogen metabolism in astrocytes: physiological, pharmacological, and pathological aspects,” in *Astrocytes: Pharmacology and Function*, ed. Murphy S., (San Diego, CA: Academic Press, Inc.), 243–265. 10.1016/b978-0-12-511370-0.50015-1

[B56] Magistretti P. J. Sorg O. Yu N. Martin J. L. Pellerin L. (1993b). Neurotransmitters regulate energy metabolism in astrocytes: implications for the metabolic trafficking between neural cells. *Dev. Neurosci.* 15 306–312. 10.1159/000111349 7805583

[B57] Magistretti P. J. Sorg O. Naichen Y. Pellerin L. de Rham S. Martin J. L. (1994). Regulation of astrocyte energy metabolism by neurotransmitters. *Ren. Physiol. Biochem.* 17 168–171. 10.1159/000173810 7518950

[B58] Mangia S. Simpson I. A. Vannucci S. J. Carruthers A. (2009). The in vivo neuron-to-astrocyte lactate shuttle in human brain: evidence from modeling of measured lactate levels during visual stimulation. *J. Neurochem.* 109(Suppl. 1), 55–62. 10.1111/j.1471-4159.2009.06003.x 19393009 PMC2679179

[B59] McIlwain H. Bachelard H. S. (1985). *Biochemistry and the Central Nervous System.* London: Churchill Livingstone.

[B60] Mosienko V. Teschemacher A. G. Kasparov S. (2015). Is L-lactate a novel signaling molecule in the brain? *J. Cereb. Blood Flow Metab.* 35 1069–1075. 10.1038/jcbfm.2015.77 25920953 PMC4640281

[B61] Mulkidjanian A. Y. Bychkov A. Y. Dibrova D. V. Galperin M. Y. Koonin E. V. (2012). Origin of first cells at terrestrial, anoxic geothermal fields. *Proc. Natl. Acad. Sci. U.S.A.* 109 E821–E830. 10.1073/pnas.1117774109 22331915 PMC3325685

[B62] Nagy K. A. Girard I. A. Brown T. K. (1999). Energetics of free-ranging mammals, reptiles, and birds. *Annu. Rev. Nutr.* 19 247–277. 10.1146/annurev.nutr.19.1.247 10448524

[B63] Nelson S. R. Schulz D. W. Passonneau J. V. Lowry O. H. (1968). Control of glycogen levels in brain. *J. Neurochem.* 15 1271–1279. 10.1111/j.1471-4159.1968.tb05904.x 5707418

[B64] O’Dowd B. S. Gibbs M. E. Ng K. T. Hertz E. Hertz L. (1994). Astrocytic glycogenolysis energizes memory processes in neonate chicks. *Brain Res. Dev. Brain Res.* 78 137–141. 10.1016/0165-3806(94)90018-3 8004768

[B65] Pellerin L. Magistretti P. J. (1994). Glutamate uptake into astrocytes stimulates aerobic glycolysis: a mechanism coupling neuronal activity to glucose utilization. *Proc. Natl. Acad. Sci. U.S.A.* 91 10625–10629. 10.1073/pnas.91.22.10625 7938003 PMC45074

[B66] Pellerin L. Magistretti P. J. (2012). Sweet sixteen for ANLS. *J. Cereb. Blood Flow Metab.* 32 1152–1166. 10.1038/jcbfm.2011.149 22027938 PMC3390819

[B67] Phelps C. H. (1972). Barbiturate-induced glycogen accumulation in brain. An electron microscopic study. *Brain Res.* 39 225–234. 10.1016/0006-8993(72)90797-4 5025645

[B68] Ransom B. R. Fern R. (1997). Does astrocytic glycogen benefit axon function and survival in CNS white matter during glucose deprivation? *Glia* 21 134–141. 10.1002/(sici)1098-1136(199709)21:1<134::aid-glia15>3.3.co;2-p 9298856

[B69] Ransom B. R. Stys P. K. Waxman S. G. (1990). The pathophysiology of anoxic injury in central nervous system white matter. *Stroke* 21 52–57. 2237986

[B70] Rossen R. Kabat H. Anderson J. P. (1943). Acute arrest of cerebral circulation in man. *Arch. Neurol. Psychol.* 50 510–528.

[B71] Ruminot I. Schmalzle J. Leyton B. Barros L. F. Deitmer J. W. (2019). Tight coupling of astrocyte energy metabolism to synaptic activity revealed by genetically encoded FRET nanosensors in hippocampal tissue. *J. Cereb. Blood Flow Metab.* 39 513–523. 10.1177/0271678X17737012 29083247 PMC6421254

[B72] Sonnewald U. Westergaard N. Schousboe A. (1997). Glutamate transport and metabolism in astrocytes. *Glia* 21 56–63. 10.1002/(sici)1098-1136(199709)21:1<56::aid-glia6>3.0.co;2-# 9298847

[B73] Sorg O. Pellerin L. Stolz M. Beggah S. Magistretti P. J. (1995). Adenosine triphosphate and arachidonic acid stimulate glycogenolysis in primary cultures of mouse cerebral cortical astrocytes. *Neurosci. Lett.* 188 109–112. 10.1016/0304-3940(95)11410-x 7792053

[B74] Sotelo-Hitschfeld T. Niemeyer M. I. Machler P. Ruminot I. Lerchundi R. Wyss M. T. (2015). Channel-mediated lactate release by K+-stimulated astrocytes. *J. Neurosci.* 35 4168–4178. 10.1523/JNEUROSCI.5036-14.2015 25762664 PMC6605297

[B75] Stryer L. (1995). *Biochemistry.* New York, NY: W.H. Freeman & Co.

[B76] Stys P. K. Ransom B. R. Waxman S. G. (1991). Compound action potential of nerve recorded by suction electrode: a theoretical and experimental analysis. *Brain Res.* 546 18–32. 10.1016/0006-8993(91)91154-s 1855148

[B77] Stys P. K. Waxman S. G. Ransom B. R. (1992). Ionic mechanisms of anoxic injury in mammalian CNS white matter: role of Na+ channels and Na+-Ca2+ exchanger. *J. Neurosci.* 12 430–439. 10.1523/jneurosci.12-02-00430.1992 1311030 PMC6575619

[B78] Suzuki A. Stern S. A. Bozdagi O. Huntley G. W. Walker R. H. Magistretti P. J. (2011). Astrocyte-neuron lactate transport is required for long-term memory formation. *Cell* 144 810–823. 10.1016/j.cell.2011.02.018 21376239 PMC3073831

[B79] Swanson R. A. (1992). Physiologic coupling of glial glycogen metabolism to neuronal activity in brain. *Can. J. Physiol. Pharmacol.* 70 S138–S144. 1295664 10.1139/y92-255

[B80] Swanson R. A. Choi D. W. (1993). Glial glycogen stores affect neuronal survival during glucose deprivation in vitro. *J. Cereb. Blood Flow Metab.* 13 162–169. 10.1038/jcbfm.1993.19 8417005

[B81] Tadi M. Allaman I. Lengacher S. Grenningloh G. Magistretti P. J. (2015). Learning-induced gene expression in the hippocampus reveals a role of neuron-astrocyte metabolic coupling in long term memory. *PLoS One* 10:e0141568. 10.1371/journal.pone.0141568 26513352 PMC4625956

[B82] Tekkok S. B. Brown A. M. Westenbroek R. Pellerin L. Ransom B. R. (2005). Transfer of glycogen-derived lactate from astrocytes to axons via specific monocarboxylate transporters supports mouse optic nerve activity. *J. Neurosci. Res.* 81 644–652. 10.1002/jnr.20573 16015619

[B83] Thorens B. Mueckler M. M. (2010). Glucose transporters in the 21st Century. *Am. J. Physiol. Endocrinol. Metab.* 298 E141–E145. 20009031 10.1152/ajpendo.00712.2009 PMC2822486

[B84] Tian G. F. Baker A. J. (2000). Glycolysis prevents anoxia-induced synaptic transmission damage in rat hippocampal slices. *J. Neurophysiol.* 83 1830–1839. 10.1152/jn.2000.83.4.1830 10758095

[B85] Tsacopoulos M. Veuthey A. L. Saravelos S. G. Perrottet P. Tsoupras G. (1994). Glial cells transform glucose to alanine, which fuels the neurons in the honeybee retina. *J. Neurosci.* 14 1339–1351. 10.1523/jneurosci.14-03-01339.1994 8120629 PMC6577565

[B86] Tymoczko J. L. Berg J. M. Stryer L. (2015). *Biochemistry: A Short Course.* New York, NY: W.H. Freeman and Co.

[B87] Vilchez D. Ros S. Cifuentes D. Pujadas L. Valles J. Garcia-Fojeda B. (2007). Mechanism suppressing glycogen synthesis in neurons and its demise in progressive myoclonus epilepsy. *Nat. Neurosci.* 10 1407–1413. 10.1038/nn1998 17952067

[B88] Voet D. Voet J. G. (2011). *Biochemistry.* Hoboken, NJ: Wiley & Sons, Inc.

[B89] Wang S. S. Shultz J. R. Burish M. J. Harrison K. H. Hof P. R. Towns L. C. (2008). Shaping of white matter composition by biophysical scaling constraints. *J. Neurosci.* 28 4047–4056. 18400904 10.1523/JNEUROSCI.5559-05.2008 PMC2779774

[B90] Wender R. Brown A. M. Fern R. Swanson R. A. Farrell K. Ransom B. R. (2000). Astrocytic glycogen influences axon function and survival during glucose deprivation in central white matter. *J. Neurosci.* 20 6804–6810. 10.1523/jneurosci.20-18-06804.2000 10995824 PMC6772835

[B91] Whatley S. A. Hall C. Lim L. (1981). Hypothalamic neurons in dissociated cell culture: the mechanism of increased survival times in the presence of non-neuronal cells. *J. Neurochem.* 36 2052–2056. 10.1111/j.1471-4159.1981.tb10833.x 6453956

[B92] Yang X. Hamner M. A. Brown A. M. Evans R. D. Ye Z. C. Chen S. (2014). Novel hypoglycemic injury mechanism: N-methyl-D-aspartate receptor-mediated white matter damage. *Ann. Neurol.* 75 492–507. 10.1002/ana.24050 24242287

[B93] Zhao Y. Zhang Q. Shao X. Ouyang L. Wang X. Zhu K. (2017). Decreased glycogen content might contribute to chronic stress-induced atrophy of hippocampal astrocyte volume and depression-like behavior in rats. *Sci. Rep.* 7:43192. 10.1038/srep43192 28233800 PMC5324119

